# Effect of Sopoongsan on Skin Inflammation and Hyperlocomotion in Socially Isolated Mice with Atopic Dermatitis

**DOI:** 10.1155/2022/3323201

**Published:** 2022-09-16

**Authors:** Ly Thi Huong Nguyen, Min-Jin Choi, Heung-Mook Shin, In-Jun Yang

**Affiliations:** ^1^Department of Physiology, College of Korean Medicine Dongguk University, Gyeongju 38066, Republic of Korea; ^2^R&D Center, Etnova Therapeutics Corporation, 124 Sagimakgol-ro, Jungwon-gu, Gyeonggi-do 13207, Republic of Korea

## Abstract

Psychological stress is a major exacerbating factor of atopic dermatitis (AD), a chronic inflammatory skin disease. Sopoongsan (SPS), a traditional herbal formula, has been indicated for the treatment of various skin disorders, including AD. This study investigated the effects of SPS on a 2,4-dinitrochlorobenzene- (DNCB-) induced AD mice model exposed to social isolation (SI) stress. The severity of the AD symptoms and behavioral abnormalities was evaluated. SPS reduced the clinical skin score as evaluated with the SCORing Atopic Dermatitis (SCORAD) index and suppressed the cutaneous infiltration of T-lymphocyte cells, mast cells, and eosinophils in SI-AD mice. The SPS treatment decreased the total distance and mean speed and increased resting time in the open field test (OFT) for these mice. In addition, the time spent in the social zone in the social interaction test also improved when SPS treatment was given. The levels of tumor necrosis factor-alpha (TNF-*α*) and interleukin-6 (IL-6) in the prefrontal cortex (PFC) in the SI-AD mice were reduced by the oral administration of SPS. HaCaT and BV2 cells were used for the *in vitro* experiments. The pretreatment with SPS reduced the protein levels of the thymus and activation-regulated chemokine (TARC) and macrophage-derived chemokine (MDC) in the HaCaT cells stimulated with TNF-*α* and interferon-gamma (IFN-*γ*) (TI). SPS also suppressed TNF-*α* and IL-6 secretion in lipopolysaccharide- (LPS-) stimulated BV2 cells. These results imply that SPS could be a promising candidate for the treatment of AD in patients under stress conditions and at risk of exacerbation.

## 1. Introduction

Atopic dermatitis (AD) is an inflammatory skin disorder, which presents as severe erythematous and itchy papules in the acute phase and lichenification and dry, thickened skin lesions in the chronic phase [1]. AD occurs due to complex interactions between environmental stimuli, the skin barrier, and the immune system [2]. Recent studies have linked psychological stress to the development or exacerbation of AD. Patients with stress-exacerbated AD were found to have elevated levels of cortisol, the “stress hormone,” and there was a positive correlation between the cortisol levels and disease severity [3]. Additionally, AD patients with psychological stress experienced more severe itching and scratching symptoms than those without stress [4]. Numerous studies have been undertaken to understand the pathophysiology of psychological stress in the development of AD and its role in exacerbating preexisting AD. Stress is believed to trigger a shift from the T helper 1 (Th1) to Th2 response, eventually enhancing the development of AD [5]. Moreover, psychological stress can stimulate mast cells to release various inflammatory chemokines and cytokines through the activation of the hypothalamic-pituitary-adrenal (HPA) axis and secretion of neuropeptides [6]. Further, high levels of cortisol increase the secretion of inflammatory molecules from keratinocytes and impair the skin barrier, exacerbating AD symptoms [7].

Uncontrolled or persistent psychological stress is known to alter brain function, and it can result in neuropsychiatric disorders [8]. On the other hand, patients with AD appear to be at higher risk of multiple neuropsychiatric disorders. These include attention-deficit/hyperactivity disorder (ADHD) and speech disorders specifically in children and depression, headaches, and seizures in the overall population. Psychological stress could worsen not only the symptoms of AD, but also neuropsychiatric comorbidities in AD patients [9–11]. Studies show that psychological stress alters neural connections and neurotransmitter actions in the prefrontal cortex (PFC) in AD patients [12, 13]. Hence, people with AD are more susceptible to psychological stress as it has a direct impact on both the skin and neuropsychiatric health. Current medications for the management of AD include topical corticosteroids, which have an anti-inflammatory effect and selective serotonin reuptake inhibitors (SSRIs) for the management of stress-related symptoms. However, several adverse effects due to their misuse have often been reported [14, 15]. This necessitates the development of safer and more effective therapeutic approaches that can simultaneously address the management of both atopic dermatitis and stress-related disorders.

Traditional herbal medicines contain multiple ingredients with varying medicinal properties. Such medicines could effectively address comorbid conditions in addition to the disease of primary interest [16]. Sopoongsan (SPS) is a traditional herbal medicine used for the treatment of various skin diseases in Korea. It has been mentioned in the ancient Korean traditional medicine book, Dongui Bogam. SPS contains 12 medicinal herbs: Schizonepetae Spica*, Glycyrrhizae Radix, Ginseng Radix,* Poria Sclerotium*, Bombycis Corpus cum Batryticatus,* Ligustici Rhizoma*, Saposhnikovia Radix, Agastachis Herba, Cicadae Periostracum, Osterici Radix,* Citri Pericarpium*, and Magnoliae Cortex*. Treatment with SPS has been reported to reduce ear thickness and inflammatory cytokine levels in a mouse model of contact hypersensitivity [17]. Additionally, SPS has been shown to suppress the immune response and demonstrate antiallergic activity in mice and rats [18]. The oral administration of SPS alleviated swelling and reduced the serum levels of immunoglobulin E (IgE) and histamine in a rat model of type I allergic dermatitis [19].

Interestingly, various components of SPS also possess beneficial effects on psychological stress and neuropsychiatric diseases. *Glycyrrhizae Radix, Ginseng Radix, and* Poria Sclerotium have been shown to attenuate stress-induced behavioral changes and regulate the expression of neurotransmitters in mice [20–22]. *Cicadae Periostracum, Bombycis Corpus cum Batryticatus, and Magnoliae Cortex* have demonstrated neuroprotective effects in the *in vivo* and *in vitro* models [23–25]. Similarly, Ligustici Rhizoma*, Saposhnikovia Radix, and Agastachis Herba* are the main ingredients of herbal medicines, namely, Jiawey Siwu, Xiao-xu-ming decoction, and Sunghyangjungisan, respectively, which have demonstrated neuroprotective effects [26–28]. Recently, the antineuroinflammatory activity of *Citri pericarpium* was also demonstrated on lipopolysaccharide-activated BV-2 microglial cells [29]. Based on the above data, we aimed to investigate the efficacy and underlying mechanism of SPS on skin symptoms and neuropsychiatric comorbidities in AD mice exposed to psychological stress.

## 2. Materials and Methods

### 2.1. Preparation of the Sopoongsan (SPS) Extract

SPS was obtained from the Kyung Hee University Hospital (Seoul, South Korea).  Table 1 lists the composition of SPS. The SPS extract was prepared with 70% aqueous ethanol (1 : 10, w/v) at 70°C for 3 h. The fluid extract was collected by filtering through filter paper (Whatman Grade 2, Whatman International, Maidstone, UK). The extract was then evaporated using a vacuum evaporator (EYELA, Tokyo Rikakikai, Tokyo, Japan) and cryodesiccated in a freeze dryer (FD8508S, Ilshin, Busan, South Korea). The obtained material (yield 7.88%, w/w) was dissolved in dimethyl sulfoxide (DMSO), sterilized using a syringe filter (0.22 *μ*m, Sartorius, Goettingen, Germany), and kept at −20°C for further use.

### 2.2. High-Performance Liquid Chromatography (HPLC)

The levels of astilbin (1), hesperidin (2), acacetin (3), senkyunolide A (4), imperatorin (5), magnolol (6), glycyrrhizic acid (7), and pulegone (8) were evaluated by HPLC (Agilent 1290 series, Santa Clara, CA). The HPLC of the SPS extract and standard compounds was conducted at the Korea Basic Science Institute (KBSI) (Seoul, Korea). The samples (10 *μ*l) were injected into a Kinetex C18 column (4.6 × 250 mm, 5 *μ*m, Phenomenex) with a guard column (UHPLC C18, AJ0-8768, Phenomenex). The mobile phases included (A) 0.1% phosphoric acid and (B) acetonitrile. The flow rate was 0.9 ml/min. The solvent gradient was set to the following: 10 to 90% (B) for 25 min and equilibration for 5 min. Astilbin, hesperidin, acacetin, senkyunolide A, imperatorin, and magnolol were detected at 210 nm. Glycyrrhizic acid and pulegone were detected at 254 nm. The column temperature was 35°C. The components of SPS were quantified from standard curves.

### 2.3. Animal Experiment

**Figure 1:**
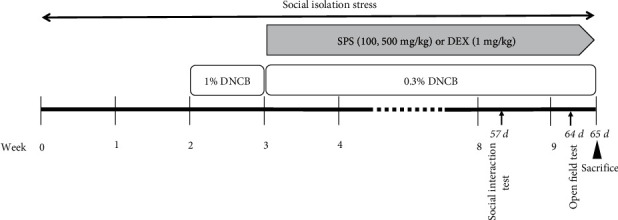
Animal experiment schedule for the induction of AD, SI stress, and SPS treatment. AD: atopic dermatitis; SPS: Sopoongsan; SI: social isolation.

BALB/c mice (male, three weeks old) were obtained from Koatech (Seoul, South Korea). All procedures were conducted according to the guidelines of the Institutional Animal Care and Use Committee of Dongguk University (IACUC-2020-05). 2,4-Dinitrochlorobenzene (DNCB) was used to induce AD-like skin lesions in the BALB/c mice as previously described [30, 31]. The animals were divided randomly into six groups as follows: the NC group (normal control mice), SI-AD group (DNCB-treated mice exposed to social isolation (SI) stress), SPS100 group (SI-AD mice treated with SPS 100 mg/kg/day), SPS500 group (SI-AD mice treated with SPS 500 mg/kg/day), and the DEX group (SI-AD mice treated with dexamethasone 1 mg/kg/day). All mice were acclimated for one week before the experiment.  Figure 1 shows the experimental schedule. The SI stress was induced by housing individual mice in small acrylic cages (10 × 10 × 14 cm). The mice were subjected to SI stress for two weeks before the DNCB treatment (except for the NC and AD groups). The DNCB was dissolved in an acetone/olive oil solution (3 : 1) for topical application. During the sensitization period (first week after SI stress), 200 *μ*l of 1% DNCB was applied to the skin over the back of the mouse as previously described [32]. The following week, 0.3% of DNCB (200 *μ*l) was applied. This maintained the AD lesions for the next six weeks. SPS (100 or 500 mg/kg) or DEX (1 mg/kg) was administered orally once daily for six weeks. All mice were sacrificed using isoflurane. The severity of the skin symptoms was assessed using a modified SCORAD (SCORing Atopic Dermatitis) index, which assesses the severity of AD based on four criteria (erythema, dryness, edema, and excoriation) on a scale of 0–3 (0, none; 1, mild; 2, moderate; 3, severe) [33]. The body and spleen weights were recorded. The skin, blood, and brain samples were collected for further experiments.

### 2.4. Open Field Test (OFT)

An OFT was conducted to examine the effects of SPS on the locomotion of the SI-AD mice as previously described [33]. All the mice were stabilized in the test room for 2 h before the OFT. Each mouse was placed in a black box (30 × 30 × 30 cm) for 15 min. All boxes were cleaned with 70% ethanol before use and after every test to eliminate any odors. The total distance traveled (cm), time in the center zone (s), and resting times were assessed using the SMART V3.0 software (Panlab, Barcelona, Spain).

### 2.5. Social Interaction Test

A social interaction test was conducted to investigate the social behavior in the SI-AD mice as previously described with modifications [34]. All the mice were acclimatized in the test room for 2 h before the social interaction test. Each mouse was placed in a black box (30 × 30 × 30 cm) containing a small cylinder wire cage (diameter: 8 cm, height: 10 cm). All boxes and cages were cleaned with 70% ethanol before use and after every test to eliminate odors. Each mouse was placed in the test box for 3 min for habituation. An unfamiliar mouse (same strain, sex, and age) was then put in the small cage. The social zone was defined as a 4 cm wide area surrounding the interaction cage. The number of entries into the social zone, time spent in the social zone (s), total distance traveled (cm), and resting time (*s*) were recorded using SMART V3.0 software (Panlab, Barcelona, Spain).

### 2.6. Histological and Immunohistochemical (IHC) Analysis

Skin tissues were fixed in 4% formaldehyde and embedded in paraffin. The skin sections (5 *μ*m-thick) were stained with hematoxylin and eosin (H&E), toluidine blue, or Congo red to evaluate epidermal thickness, number of mast cells, and number of eosinophils, respectively. For IHC staining, the sections were incubated with the anti-CD3 antibody overnight at 4°C, followed by the horseradish peroxidase- (HRP-) conjugated secondary antibody for 1 h at room temperature, and visualized using an aminoethyl carbazole (AEC) chromogen kit (Sigma-Aldrich, St. Louis, MO, USA). The samples were examined using a Lionheart FX microscope with Gen5 imaging software (BioTek Instruments, Winooski, VT, USA). The epidermal thickness, number of mast cells and eosinophils, and intensity of CD3 were evaluated in at least three random sites for each sample.

### 2.7. Serum Levels of Glutamic Pyruvic Transaminase/Alanine Transaminase (GPT/ALT) and Glutamic Oxaloacetic Transaminase/Aspartate Transaminase (GOT/AST)

To examine whether SPS can cause hepatotoxicity, the GPT/ALT and GOT/AST levels in the serum were evaluated using Fuji DRI-CHEM slide GPT/ALT and GOT/AST kits (Fujifilm, Tokyo, Japan), according to the manufacturer's protocols.

### 2.8. Cell Culture and Treatments

The HaCaT cells (a human keratinocyte cell line) and BV2 cells (a murine microglial cell line) were grown in Dulbecco's Modified Eagle Medium (DMEM) high glucose (Welgene Inc., Gyeongsangbuk-do, South Korea) supplemented with 10% fetal bovine serum (FBS) (Merck KGaA, Darmstadt, Germany), and 1% antibiotics (ThermoFisher Scientific, Waltham, MA), at 37°C in a 5% CO_2_ humidified incubator. The HaCaT cells and BV2 cells were preincubated with SPS (10, 50, or 100 *μ*g/ml) or DEX (10 *μ*M) for 1 h and treated with TI (tumor necrosis factor-alpha (TNF-*α*) and interferon-gamma (IFN-*γ*), 10 ng/ml each) or lipopolysaccharide (LPS, 1 *μ*g/ml), respectively, for 24 h.

### 2.9. MTT (3-(4,5-Dimethylthiazol-2-yl)-2,5-diphenyltetrazolium Bromide) Assays

The effects of the SPS on the viability of the HaCaT and BV2 cells were examined using MTT assays. The cells were seeded at a density of 5 × 10^4^ cells/well to 96-well plates. After 24 h, the cells were treated with SPS (10, 50, 100, and 500 *μ*g/ml) for 24 h. After treatment, the culture medium was removed, and then, 100 *μ*l fresh medium and 10 *μ*l of MTT reagent (5 mg/ml) (Sigma-Aldrich, St. Louis, MO, USA) were added to each well. The plate was incubated at 37°C for 4 h. The medium was removed, and 50 *μ*l DMSO was added to each well and incubated for 30 min. Absorbances were measured at 570 nm using a microplate reader (Tecan, Männedorf, Switzerland).

### 2.10. Enzyme-Linked Immunosorbent Assay (ELISA)

The levels of inflammatory cytokines were evaluated using ELISA kits according to the manufacturer's protocols, including interleukin (IL)-6 and TNF-*α* levels in the cultured media from the BV2 cells, thymus, and activation-regulated chemokine (TARC), and macrophage-derived chemokine (MDC) levels in the cultured media from HaCaT cells, and the levels of TNF-*α*, IL-6, and IL-1*β* in the PFC. The mouse IL-6, TNF-*α*, and IL-1*β* ELISA kits were purchased from LABISKOMA (Seoul, South Korea). The human TARC and MDC ELISA kits were procured from R&D Systems (Minneapolis, MN). The absorbance at 450–550 nm was assessed using a microplate reader (Tecan, Männedorf, Switzerland).

### 2.11. Statistical Analysis

All experiments were conducted at least three times independently. The data represent the mean ± standard deviation (SD), and *p* values < 0.05 were considered statistically significant using a two-tailed unpaired Student's *t*-test. Correlations between two parameters were analyzed using Pearson's correlation coefficient (*r*).

## 3. Results

### 3.1. SPS Alleviated Clinical Symptoms of AD in the SI-AD Mice

**Figure 2:**
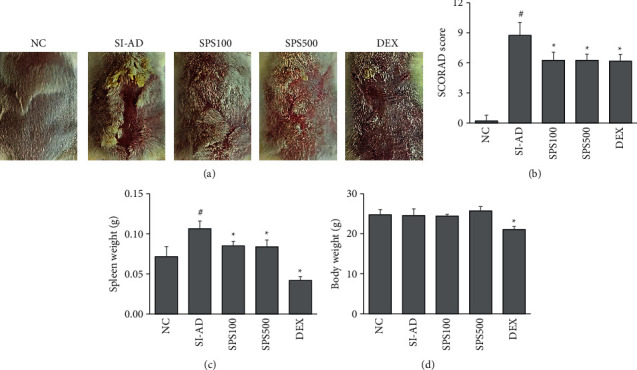
Effects of SPS on AD-like symptoms in the SI-AD mice. (a) Representative images of skin lesions from six groups. (b–d) The SCORAD scores, spleen weights, and body weights were measured. Data are expressed as means ± SDs (*n* = 6 per experiment). ^#^*p* < 0.05, vs. the NC group; ^*∗*^*p* < 0.05, vs. the SI-AD group (unpaired Student's *t*-test). SPS: Sopoongsan; AD: atopic dermatitis; NC: normal control; SI-AD: atopic dermatitis mice exposed to social isolation stress.

Studies have demonstrated that the SI stress model in mice is useful for the evaluation of psychological stress in AD [35]. Our previous study demonstrated that SI stress could exaggerate dermatitis and induce hyperactivity in DNCB-induced AD mice [33]. Hence, this study examined the effects of SPS in the SI-AD mouse model. SPS ameliorated the severity of skin symptoms, such as scaling and erythema, compared to the SI-AD group (Figure 2(a)). The SI-AD mice showed a remarkably higher SCORAD index, which was significantly ameliorated by SPS treatment at doses of 100 and 500 mg/kg (*p* < 0.05) (Figure 2(b)). The effects of SPS were comparable with the effects seen with the positive control drug (DEX). Moreover, treatment with SPS decreased the spleen weight significantly (*p* < 0.05) (Figure 2(c)), without affecting the body weight (Figure 2(d)). In contrast, the DEX treatment decreased both the spleen weight and the body weight significantly (*p* < 0.05) (Figures 2(c) and 2(d)). The oral administration of SPS did not have any hepatotoxic effects, and the SPS treatment resulted in decreased serum levels of GPT/ALT and GOT/AST in SI-AD mice (*p* < 0.05) (Table 2).

### 3.2. SPS Ameliorated Histopathological Symptoms in the SI-AD Mice

**Figure 3:**
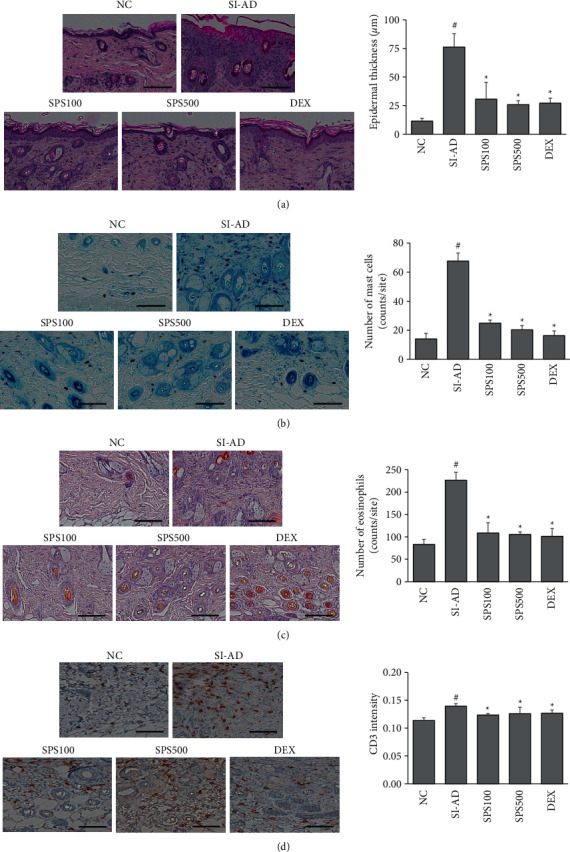
Effects of SPS on epidermal thickening and dermal infiltration of immune cells in the SI-AD mice. Representative images of H&E: (a) toluidine blue, (b) Congo red staining, (c) or immunohistochemical staining of CD3 (d) from each group (magnification 200 ×, scale bar 100 *μ*m). Data are expressed as means ± SDs (*n* = 3 per experiment). ^#^*p* < 0.05, vs. the NC group; ^*∗*^*p* < 0.05, vs. the SI-AD group (unpaired Student's *t*-test). SPS: Sopoongsan; H&E: hematoxylin and eosin; NC: normal control; SI-AD: atopic dermatitis mice exposed to social isolation stress.

Epidermal hyperplasia and dermal infiltration of inflammatory cells were typical histopathological symptoms in the DNCB-induced mouse model of AD [36,37]. H&E staining showed that SI-AD mice exhibited epidermal thickening, which was significantly reduced by the SPS treatment (*p* < 0.05) (Figure 3(a)). Dermal infiltration of mast cells and eosinophils was observed by toluidine blue and Congo red staining. Oral treatment with SPS significantly decreased the number of infiltrated mast cells and eosinophils in the AD-like skin lesions, compared to the SI-AD group (*p* < 0.05) (Figures 3(b) and 3(c)). IHC staining showed that SPS significantly lowered the expression of CD3, a T cell biomarker in the skin lesions of the SI-AD mice (*p* < 0.05) (Figure 3(d)).

### 3.3. SPS Improved Behavioral Abnormalities in the SI-AD Mice

**Figure 4:**
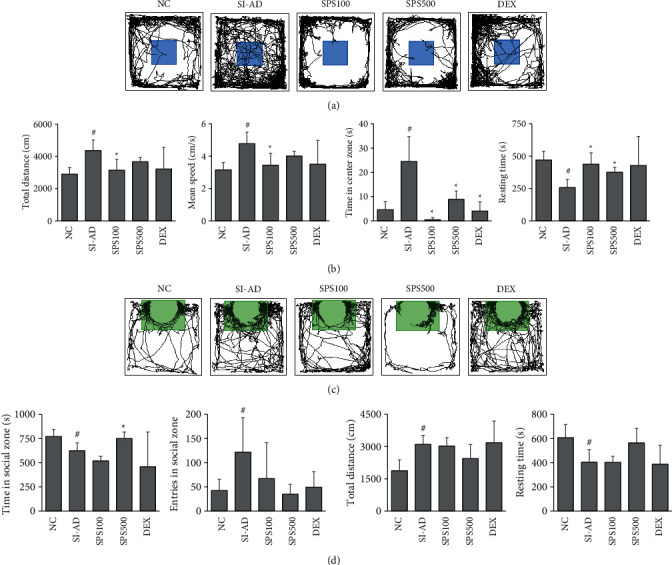
Effects of SPS on the behavioral abnormalities in the SI-AD mice. (a) Representative tracking paths in the OFT from each group. The blue areas indicate the center zone. (b) Total distances (cm), mean speed (cm/s), time in center zone (cm), and resting time in a 15-min OFT session. (c) Representative tracking paths in the social interaction test. The green areas indicate the social zone. (d) Time in the social zone, entries in the social zone, total distance (cm), and resting time in the social interaction test. Data are expressed as means ± SDs (*n* = 4–6 per experiment). ^#^*p* < 0.05, vs. the NC group; ^*∗*^*p* < 0.05, vs. the SI-AD group (unpaired Student's *t*-test). SPS: Sopoongsan; OFT: open field test; NC: normal control; SI-AD: atopic dermatitis mice exposed to social isolation stress.

The next step was to determine whether SPS could treat neurobehavioral disorders worsened by psychological stress in an AD mouse model. For this purpose, we conducted OFT and social interaction tests. The total distance, mean speed, and time spent in the central zone in the OFT were significantly higher, and the resting time was remarkably lower in the SI-AD mice compared to the NC mice (*p* < 0.05) (Figures 4(a) and 4(b)). In contrast, the SPS treatment (100 mg/kg) reduced the total distance and mean speed in the SI-AD mice significantly (*p* < 0.05) (Figure 4(b)). This was not seen in the DEX-treated mice. Treatment with SPS (100 and 500 mg/kg) also normalized the time in the center zone and resting time in the OFT, compared to the SI-AD mice (*p* < 0.05) (Figure 4(b)). In the social interaction test, the SI-AD mice showed a reduced time spent in the social zone and an increased number of entries into the social zone (*p* < 0.05) (Figures 4(c) and 4(d)). This might show one-sided social behavior induced by the SI-AD condition. Similar to OFT, in the social interaction test, the total distance was significantly higher, and resting time was significantly lower in the SI-AD group (*p* < 0.05). On the other hand, SPS treatment could increase the time spent in the social zone (*p* < 0.05) but had no effect on entries in the social zone, total distance, and resting time in the social interaction test.

### 3.4. SPS Reduced Neuroinflammation in the SI-AD Mice

**Figure 5:**
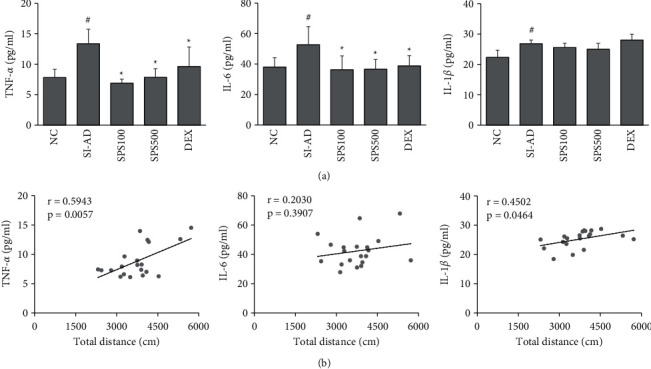
Effects of SPS on neuroinflammation in the SI-AD mice. (a) Levels of cytokines TNF-*α*, IL-6, and IL-1*β* in the prefrontal cortex were measured using ELISA kits. (b) Correlations between the total distance in the OFT and the levels of TNF-*α*, IL-6, and IL-1*β* in the prefrontal cortex were analyzed using Pearson's correlation coefficient (*r*). Data are expressed as means ± SDs (*n* = 6 per experiment). ^#^*p* < 0.05, vs. the NC group; ^*∗*^*p* < 0.05, vs. the SI-AD group (unpaired Student's *t*-test). SPS: Sopoongsan; OFT: open field test; NC: normal control; SI-AD: atopic dermatitis mice exposed to social isolation stress; TNF- *α*: tumor necrosis factor-alpha; IL: interleukin.

Neuroinflammation plays an important role in psychological stress-induced behavioral changes [38]. Research has indicated that individuals with ADHD have disrupted PFC structure and function [39]. Here, we investigated the effects of SPS on the levels of inflammatory cytokines in the PFC in SI-AD mice. The results showed that SI-AD mice had significantly higher levels of TNF-*α*, IL-6, and IL-1*β* in the PFC than the NC mice (*p* < 0.05) (Figure 5(a)). On the other hand, SPS administration decreased the production of TNF-*α* and IL-6 significantly (*p* < 0.05). Moreover, the total distance in the OFT had positive correlations with the levels of TNF-*α* (*r* = 0.5943, *p*=0.0057) and IL-1*β* (*r* = 0.4502, *p*=0.0464) in the PFC (Figure 5(b)), suggesting that the effects of SPS on behavior might be through antineuroinflammatory activities in PFC.

### 3.5. SPS Suppressed the Inflammatory Response in HaCaT Keratinocytes and BV2 Microglial Cells

**Figure 6:**
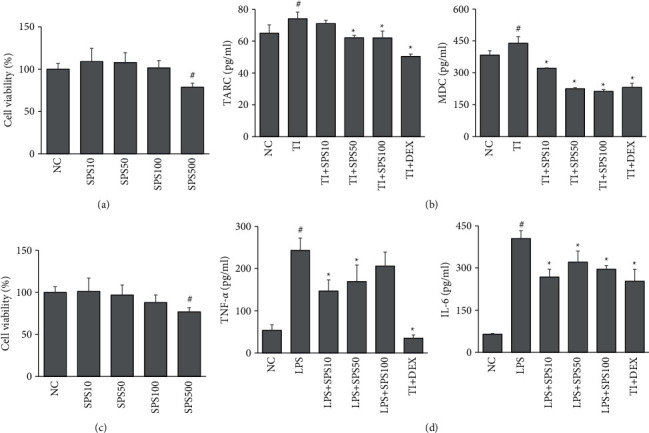
Effects of SPS on the inflammatory response in HaCaT keratinocytes and BV2 microglial cells. (a) HaCaT cells were incubated with SPS (10, 50, 100, and 500 *μ*g/ml) for 24 (h) Effects of SPS on the viability of HaCaT cells were assessed using MTT assays. (b) HaCaT cells were preincubated with SPS (10, 50, and 100 *μ*g/ml) for 1 h and stimulated with TI (TNF-*α* and IFN-*γ*, 10 ng/ml each) for 24 h The levels of MDC and TARC in the cell culture media were measured using ELISA kits. (c) BV2 cells were incubated with SPS (10, 50, 100, and 500 *μ*g/ml) for 24 h Effects of SPS on the viability of BV2 cells were assessed using MTT assays. (d) BV2 cells were pre-incubated with SPS (10, 50, 100 *μ*g/ml) for 1 h and stimulated with LPS (1 *μ*g/ml) for 24 h The levels of TNF-*α* and IL-6 in cell culture media were measured using ELISA kits. Data are expressed as means ± SDs (*n* = 3). ^#^*p* < 0.05, vs. NC; ^*∗*^*p* < 0.05, vs. TI-treated cells, ^*∗*^*p* < 0.05, vs. LPS-treated cells (unpaired Student's *t*-test). SPS: Sopoongsan; LPS: lipopolysaccharide; TNF- *α*: tumor necrosis factor-alpha; IL: interleukin; MDC: macrophage-derived chemokine; TARC: thymus and activation-regulated chemokine.

We used HaCaT keratinocytes and BV2 microglial cells as *in vitro* models to understand the mechanism underlying the action of SPS. Keratinocytes are the primary cells of the epidermis and play a significant role in skin inflammation in AD [40]. Microglial cells act as immune cells in the brain and regulate neuroinflammatory responses [41]. The effects of SPS on the viability of HaCaT and BV2 cells were examined using MTT assays. The results showed that SPS at concentrations of 10, 50, and 100 *μ*g/ml did not have any cytotoxicity on the HaCaT and BV2 cells (Figure 6(a) and 6(c)). Hence, these concentrations were used to examine the anti-inflammatory effects of SPS.  Figure 6(b) shows that pretreatment with SPS (50 and 100 *μ*g/ml) downregulated the TI-induced production of inflammatory chemokines, MDC, and TARC significantly (*p* < 0.05), while 10 *μ*g/ml of SPS only reduced the MDC levels (*p* < 0.05). In the LPS-stimulated BV2 cells, the elevated levels of TNF-*α* were decreased by pretreatment with SPS (10 and 50 *μ*g/ml) (*p* < 0.05), while IL-6 levels were alleviated by SPS at all three concentrations (*p* < 0.05) (Figure 6(d)).

### 3.6. Quantification of Chemical Components of SPS

**Figure 7:**
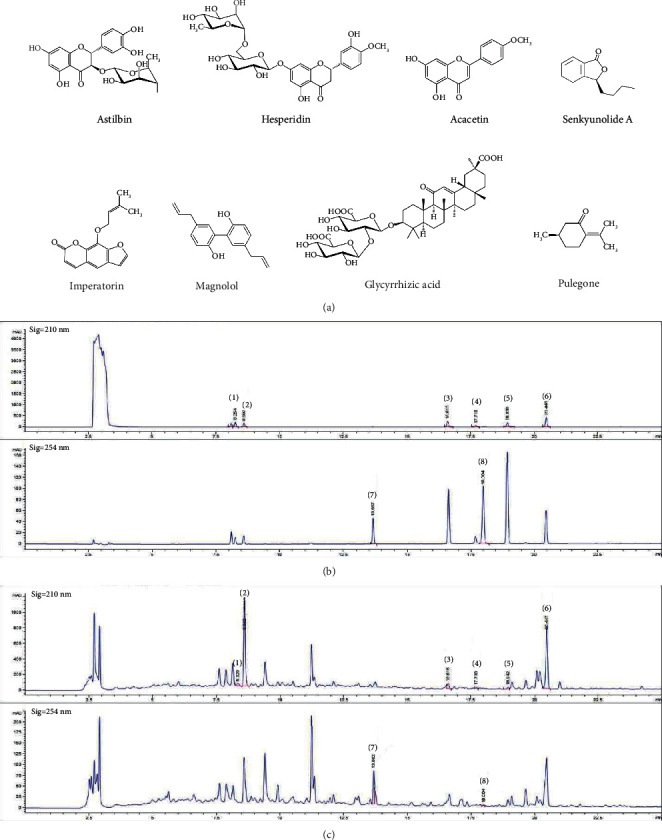
Quantification of the chemical components of SPS. (a) Structures of astilbin, hesperidin, acacetin, senkyunolide (A) imperatorin, magnolol, glycyrrhizic acid, and pulegone. HPLC chromatograms of the standard compounds (b) and SPS sample (c). Astilbin (1), hesperidin (2), acacetin (3), senkyunolide A (4), imperatorin (5), magnolol (6), glycyrrhizic acid (7), and pulegone (8). SPS: Sopoongsan; HPLC: high-performance liquid chromatography.

HPLC analysis was performed to investigate the chemical composition of SPS. Our data demonstrated that SPS contains astilbin, hesperidin, acacetin, senkyunolide A, imperatorin, magnolol, glycyrrhizic acid, and pulegone (Figure 7), the retention times and concentrations of which are presented in Table 3. The concentrations of the constituents of SPS ranged from 0.062 to 13.803 mg/g. Hesperidin, magnolol, and glycyrrhizic acid were compounds that made up the largest concentrations in the SPS extract.

## 4. Discussion

In patients with AD, psychological stress not only worsens the AD symptoms, but could aggravate preexisting neuropsychiatric comorbidities or lead to psychiatric conditions resulting in poorer patient outcomes as well. Treatment to alleviate the stress is, therefore, essential for the comprehensive management of AD [12, 13, 35, 42]. Conventional agents such as topical corticosteroids are not effective in treating symptoms induced by psychological stress such as chronic scratch dermatitis. There is, thus, a need for more effective, targeted therapies that can act on the neuroimmune factors as well as the skin [35]. The purpose of this study was to determine whether a traditional herbal medicine containing multiple ingredients can normalize AD symptoms and behavioral abnormalities aggravated by psychological stress, as well as whether it can affect the pathophysiology of AD by acting on several targets including the skin, immunity, and nerves. Indeed, our results suggest that SPS treatment could alleviate the AD-like symptoms and behavioral abnormalities as observed in the SI-AD mouse model. The effects of SPS were comparable to DEX, a common corticosteroid used for inflammatory skin diseases, with no apparent toxicity. One crucial immune organ in the body is the spleen, and an enlarged spleen is suggestive of inflammatory disease [43]. In the present study, the fact that the splenomegaly in the SI-AD group was inhibited by SPS indicated a potential role of SPS in the regulation of inflammatory responses.

AD is an immune-driven skin disease, and inflammatory cell infiltration into skin lesions is a hallmark of the condition [44]. T-cells play a vital role in the development and progression of AD [45]. In the early phase of AD, Th2 lymphocytes are the most significant infiltrates in skin lesions that secrete various cytokines, such as IL-4, IL-5, IL-10, and IL-13 to trigger a type 2 inflammatory response [46,47]. In contrast, in the later phase of AD, Th1 cell activation is more predominant with the elevated expression of IFN-*γ* and TNF-*α* [48]. The Th1/Th2-derived cytokines contribute to the skin-homing of other inflammatory cells and activation of keratinocytes, resulting in chronic cutaneous inflammation and skin barrier disruption in AD [46]. In the current study, the SPS treatment decreased the expression of CD3, a typical biomarker of T cells, which was elevated in the skin lesions of the SI-AD mice. This suggests that SPS suppressed the infiltration of T-cells to improve AD symptoms in this mouse model. This result is in sync with a previous study, wherein the herbal mixture extract KM110329 reduced the expression of CD3 in skin lesions through its inhibitory effects on the infiltration of inflammatory cells [49].

Apart from T cells, mast cells and eosinophils also play critical roles in the pathogenesis of AD [50]. The number of mast cells infiltrating the dermis increases in the chronic phase of AD [51]. Mast cells can produce numerous proinflammatory mediators, such as TNF-*α*, prostaglandin D2, and histamine, which trigger the differentiation of naïve T cells into Th1/Th2 cells and activate keratinocytes to secrete inflammatory chemokines and cytokines [50]. Similarly, eosinophils are also ubiquitous in skin lesions from AD patients and show a positive correlation with the severity of AD [52]. IL-5 and granulocyte-macrophage colony-stimulating factor (GM-CSF) secreted by Th2 cells and mast cells help activate eosinophils, and activated eosinophils, in turn, can produce a variety of proinflammatory molecules to maintain skin inflammation [53]. A previous report indicated that treatment with SSC201, a herbal formula, attenuated AD severity and reduced the number of mast cells as well as eosinophils in DNCB-induced NC/Nga mice [54]. In the present study, the elevated numbers of mast cells and eosinophils that infiltrated the dermis of the SI-AD mice were reversed significantly by the oral administration of SPS, indicating the immune-suppressive function of SPS for AD treatment.

Keratinocytes are the primary cells of the epidermis that participate directly in the pathogenesis of AD by regulating the immune response in the skin [40]. Specific stimuli induce keratinocytes to produce a range of inflammatory molecules for the homing of immune cells in AD skin lesions. TARC and MDC are expressed strongly by keratinocytes and are involved in the recruitment of Th2 lymphocytes [55]. In patients with AD, the levels of TARC and MDC, as well as their receptor C-C chemokine receptor type 4 (CCR4), are higher than those in normal individuals. The expression of these markers also has a positive correlation with the severity of AD [56–58]. Moreover, TARC has been shown to induce keratinocyte proliferation, leading to epidermal hyperplasia in AD lesions [59]. In this study, TI-stimulated HaCaT cells were used as an *in vitro* model of AD with typically elevated production of MDC and TARC, as previously described [60]. We found that pretreatment with SPS decreased the secretion of these inflammatory chemokines significantly. This result aligns with a previous study that reported the inhibitory effects of a traditional herbal formula Jakyakgamcho-tang on skin inflammation by suppressing TARC and MDC production in keratinocytes [61]. In addition, epidermal hyperplasia in SI-AD mice was also lowered by the SPS treatment. These data suggest that the antiatopic effects of SPS may occur by modulating the inflammatory response in keratinocytes.

ADHD is the most common neuropsychiatric comorbidity in children with AD [62,63]. A combination of neurological control and treatment of AD symptoms could provide better therapeutic outcomes in these AD patients. Previous studies have suggested that SI stress could induce ADHD symptoms, including hyper-locomotion, spatial attention deficit, and aggressive behavior in rodents [64,65]. In this study, SI-AD mice exhibited increases in the total distance traveled and time spent in the center zone in the OFT, indicating hyperlocomotion behavior in this mouse model. Overall, these findings are directly in line with our previous findings that demonstrated the hyperactivity symptoms of AD mice exposed to SI stress [33]. In addition, hyperlocomotion in the SI-AD mice was also demonstrated in the social interaction test with an increase in the traveling distance, as well as a decrease in resting time. Moreover, the increased number of entries in the social zone and the decreased time spent in the social zone might suggest abnormal one-sided social behavior and depression-like behavior in the SI-AD mice [66]. Several studies have also reported that depression is a common comorbidity in children with ADHD [67–69]. These results of the present study suggest that the SI-AD mice exhibit ADHD symptoms with comorbid depression, and the oral administration of SPS alleviated these behavioral abnormalities in the SI-AD mice. This indicates that SPS might be a potential candidate for the treatment of ADHD-associated AD patients.

Neuroinflammation has been reported to contribute to the development of various psychiatric disorders and neurological diseases, including ADHD [70–72]. The underlying mechanism may be through the activation of immune cells in the brain, such as microglia and astrocytes, leading to the elevated production of proinflammatory cytokines and chemokines by these cells [41,73,74]. In the current study, the levels of inflammatory mediators were upregulated in the PFC of the SI-AD mice. Moreover, the levels of the cytokines had a positive correlation with hyperactive behavior. Previous studies have demonstrated that herbal medicines and natural compounds exert beneficial effects on behavioral changes and neurological disorders through antineuroinflammatory activities and neuroprotective effects [75–78]. In this study, the oral administration of SPS reduced the levels of TNF-*α* and IL-6 significantly in the PFC, as well as in LPS-stimulated BV2 microglial cells. Hence, the effects of SPS on behavioral changes could be mediated by antineuroinflammatory activity.

HPLC showed that hesperidin, magnolol, and glycyrrhizic acid are the major components in the SPS extract. Previous studies have reported that these compounds exhibit therapeutic effects in both dermatological and neuropsychiatric diseases. In an earlier study, hesperidin alleviated the clinical severity of AD symptoms in the NC/Nga mouse model by modulating the T cell response [79]. A previous study also demonstrated the antiatopic effect of glycyrrhizic acid in DNCB-induced BALB/c mice by inhibiting mast cell activation [80]. Magnolol has been reported to exert anti-inflammatory effects in both *in vitro* and *in vivo* models resulting in beneficial effects on skin diseases [81,82]. Moreover, previous studies have suggested that hesperidin and glycyrrhizic acid could amend chronic stress-induced depression-like behavior in mice by suppressing the activity of high-mobility group box 1 (HMGB1), a common biomarker for neuroinflammation [83,84]. Magnolol also improved stress-induced behavioral changes in mice by inhibiting proinflammatory cytokine production from microglia in the PFC [85]. These findings suggest that hesperidin, magnolol, and glycyrrhizic acid could contribute to the beneficial effects of SPS on SI stress-exacerbated AD.

Commonly used topical drugs including DEX have several limitations including the development of multidrug-resistant organisms, high toxicity, and inadequate penetration into skin lesions [32,86,87]. Moreover, in the present study, unlike SPS, the DEX treatment resulted in a remarkable reduction in the body weight of SI-AD mice. SPS, on the other hand, improved the symptoms related to the clinical manifestations of dermatitis and behavioral abnormalities and did not induce any measurable toxicity or sensitization in SI-AD mice. This suggests that SPS could be a reliable and more efficacious candidate for the treatment of AD patients exposed to psychological stress.

## 5. Conclusion

Our study is the first to report that orally administered SPS could reduce aberrant behavior as well as AD-like symptoms exacerbated by psychological stress in a mouse model. SPS has been demonstrated to be particularly effective in ameliorating hyperactivity and decreased social interaction in the mice model. These symptoms are frequently seen in patients with AD. In addition to reducing skin inflammation via controlling keratinocytes, eosinophils, mast cells, and T cells, all of which are typically linked to the development of AD, SPS also appears to have an anti-inflammatory effect on the central nervous system. These findings suggest that SPS could be developed as a novel treatment for AD patients who are sensitive to psychological stress or who suffer from neuropsychiatric comorbidities related to AD.

## Figures and Tables

**Table 1:** Composition of Sopoongsan (SPS).

Schizonepetae Spica	*Nepeta tenuifolia* Benth.

Glycyrrhizae Radix	*Glycyrrhiza uralensis* Fisch.

Ginseng Radix	*Panax ginseng* C. A. Mey.

Poria Sclerotium	*Poria cocos* (Schw.) Wolf

**Table 2:** Effects of SPS on the serum levels of GPT/ALT and GOT/AST in the SI-AD mice.

Group	GOT (U/L)	GPT (U/L)
NC	60.67 ± 14.83	40.5 ± 16.34
SI-AD	69.20 ± 6.61	43.00 ± 4.24
SPS100	55.00 ± 7.51^*∗*^	30.00 ± 12.46
SPS500	42.6 ± 4.93^*∗*^	20.6 ± 6.15^*∗*^
DEX	73.4 ± 39.56	44 ± 24.67

Data represent means ± SDs (*n* = 6 per experiment). ^*∗*^*p* < 0.05 vs. the SI-AD group (unpaired Student's *t*-test). SPS: Sopoongsan; GPT/ALT: glutamic pyruvic transaminase/alanine transaminase; GOT/AST: glutamic oxaloacetic transaminase/aspartate transaminase; SI-AD: atopic dermatitis mice exposed to social isolation stress.

**Table 3:** Quantification of the chemical components of Sopoongsan (SPS).

Compound	Retention time (min)	Amount (mg/g)
Astilbin	8.326	0.612
Hesperidin	8.591	13.803
Acacetin	16.614	0.557
Senkyunolide A	17.708	0.442
Imperatorin	18.942	0.259
Magnolol	20.447	3.902
Glycyrrhizic acid	13.66	3.481
Pulegone	18.002	0.062

## Data Availability

The data used to support the findings of this study are included in the article.

## Abbreviations

DNCB: 2,4-Dinitrochlorobenzene
TARC: Thymus and activation-regulated chemokine
MDC: Macrophage-derived chemokine
LPS: Lipopolysaccharide
TNF-*α*: Tumor necrosis factor-alpha.
